# Patient-Reported Discussions on Fertility Preservation Before Early-Onset Cancer Treatment

**DOI:** 10.1001/jamanetworkopen.2024.44540

**Published:** 2024-11-12

**Authors:** Samantha R. Keller, Allison Rosen, Mark A. Lewis, Hyo K. Park, Rebecca Babyak, Jill Feldman, Fei Ye, Rajiv Agarwal, Kristen K. Ciombor, Timothy M. Geiger, Cathy Eng, Katherine J. Hunzinger, Richard H. Viskochil, Michelle K. Roach, Digna R. Velez Edwards, Michele L. Cote, Andreana N. Holowatyj

**Affiliations:** 1 Department of Medicine, Vanderbilt University Medical Center, Nashville, Tennessee; 2 American Cancer Society, Atlanta, Georgia; 3 Intermountain Healthcare, Murray, Utah; 4 Providence Saint John’s Health Center, Santa Monica, California; 5 EGFR Lung Resisters Group, Chicago, Illinois; 6 Department of Biostatistics, Vanderbilt University Medical Center, Nashville, Tennessee; 7 Vanderbilt-Ingram Cancer Center, Nashville, Tennessee; 8 Department of Surgery, Vanderbilt University Medical Center, Nashville, Tennessee; 9 Department of Exercise Science, Thomas Jefferson University, Philadelphia, Pennsylvania; 10 Department of Exercise Health Sciences, University of Massachusetts Boston, Boston; 11 Department of Obstetrics and Gynecology, Vanderbilt University Medical Center, Nashville, Tennessee; 12 Indiana University Simon Comprehensive Cancer Center, Indianapolis; 13 Vanderbilt University School of Medicine, Nashville, Tennessee

## Abstract

This cross-sectional study evaluates discussion patterns about fertility preservation options reported by patients with early-onset cancer to better understand the patient experience.

## Introduction

The early-onset cancer (ie, individuals aged 18-49 years) experience is unique because there exists a greater need to treat multiple life domains—including reproductive health—that are affected by a cancer diagnosis.^1,2^ Notwithstanding the widespread recognition of the importance of a patient–health care professional discussion to address the possibility of infertility and fertility preservation (FP) options before cancer treatment,^3^ our understanding of this patient experience remains incomplete. Herein, we evaluated FP discussion patterns as reported by 473 patients with a first primary early-onset cancer in the REACT (Reproductive Health After Cancer Diagnosis & Treatment) Study.

## Methods

REACT Study recruitment, self-administered questionnaire elements, and sources and population are described in the eMethods in Supplement 1. We limited our cross-sectional study to males (age at diagnosis, 18-49 years) and females (age at diagnosis, 18-42 years) who received their diagnosis between 2013 and 2021 and who responded yes or no to the following question^4^: “Did a healthcare professional involved in your cancer care talk with you about options to preserve your fertility (e.g., sperm banking or freezing of eggs, embryos, or ovarian tissue) before you started cancer treatment?” (eFigure in Supplement 1). Differences between characteristics by patient-reported FP discussion were examined using the χ^2^ test and the Wilcoxon rank sum test. Cochran-Armitage trend tests were used to assess trends in age at diagnosis by patient-reported FP discussion. This study followed the STROBE reporting guideline. Statistical analysis was performed from November 10, 2023, to September 11, 2024, using SAS Institute software, version 9.4. All tests were 2-sided (unless otherwise specified), with *P* < .05 considered statistically significant.

## Results

One in every 2 patients (240 of 473 [50.7%]) reported that a health care professional involved in their cancer care discussed FP options before treatment initiation (Table). The proportion of these patient-reported FP discussions differed significantly by age, pregnancy history, and marital status. Patient-reported FP discussions by cancer type are presented in the Figure. The lowest prevalence of FP discussions was reported by young patients with thyroid, lung or bronchus, ovarian, and colorectal cancers (3.6% [1 of 28], 21.0% [13 of 62], 21.4% [3 of 14], and 44.2% [42 of 95], respectively).

**Table.** Characteristics of the Study Population (REACT Study, 2021)^a^

Characteristic	Patients, total No. (%)	Patient-reported FP discussion			
		Patients, No. (%)		*P* value	*P* value for trend
		No	Yes		
No.	473 (100)	233 (49.3)	240 (50.7)	NA	NA
Age at cancer diagnosis, median (IQR), y	35 (30-40)	38 (32-41)	33 (29-37)	<.001	<.001
Sex at birth					
Female	338 (71.5)	169 (72.5)	169 (70.4)	.61	NA
Male	135 (28.5)	64 (27.5)	71 (29.6)		
Gender identity					
Female	333 (70.4)	168 (72.1)	165 (68.8)	.36	NA
Male	136 (28.8)	65 (27.9)	71 (29.6)		
Nonbinary	2 (0.4)	0	2 (0.8)		
Prefer to self-describe^b^	1 (0.2)	0	1 (0.4)		
Not reported	1 (0.2)	0	1 (0.4)		
Self-identified race and ethnicity					
Non-Hispanic White	358 (75.7)	177 (76.0)	181 (75.4)	.81	NA
Other^c^	106 (22.4)	51 (21.9)	55 (22.9)		
Not reported	9 (1.9)	5 (2.1)	4 (1.7)		
Insurance or health care coverage^d^					
No	47 (9.9)	25 (10.7)	22 (9.2)	.56	NA
Yes	425 (89.9)	207 (88.8)	218 (90.8)		
Not reported	1 (0.2)	1 (0.4)	0		
Pregnancy history (including with a partner)					
No	202 (42.7)	85 (36.5)	117 (48.8)	.005	NA
Yes	230 (48.6)	128 (54.9)	102 (42.5)		
Not reported	41 (8.7)	20 (8.6)	21 (8.8)		
Marital status^d^					
Single^e^	136 (28.8)	54 (23.2)	82 (34.2)	.006	NA
Married or living with partner	311 (65.8)	162 (69.5)	149 (62.1)		
Divorced or separated	24 (5.1)	17 (7.3)	7 (2.9)		
Not reported	2 (0.4)	0	2 (0.8)		
Education level^d^					
Less than 4-y college degree	118 (24.9)	58 (24.9)	60 (25.0)	.97	NA
College graduate or 4-y degree	172 (36.4)	85 (36.5)	87 (36.3)		
Master’s or doctoral or professional school degree	166 (35.1)	80 (34.3)	86 (35.8)		
Not reported	17 (3.6)	10 (4.3)	7 (2.9)		
Country of residence					
US	332 (70.2)	162 (69.5)	170 (70.8)	.81	NA
Other	136 (28.8)	68 (29.2)	68 (28.3)		
Not reported	5 (1.1)	3 (1.3)	2 (0.8)		
Year of cancer diagnosis					
2013	9 (1.9)	6 (2.6)	3 (1.3)	.81	.10
2014	26 (5.5)	17 (7.3)	9 (3.8)		
2015	18 (3.8)	8 (3.4)	10 (4.2)		
2016	34 (7.2)	16 (6.9)	18 (7.5)		
2017	43 (9.1)	21 (9.0)	22 (9.2)		
2018	51 (10.8)	26 (11.2)	25 (10.4)		
2019	74 (15.6)	36 (15.5)	38 (15.8)		
2020	108 (22.8)	50 (21.5)	58 (24.2)		
2021	110 (23.3)	53 (22.7)	57 (23.8)		

Abbreviations: FP, fertility preservation; NA, not applicable; REACT, Reproductive Health After Cancer Diagnosis & Treatment.

^a^ *P* value calculations do not include not reported values. *P* values for trends were calculated using a 1-sided Cochran-Armitage trend test.

^b^ One patient self-described gender identity as a genderqueer trans man.

^c^ Other racial and ethnic groups included patients who self-identified as American Indian or Alaska Native; Asian or Asian American; Hispanic, Spanish, or Latino/a/x; non-Hispanic Black; non-Hispanic Middle Eastern and North African; multirace or mixed; and other, not otherwise specified.

^d^ At the time of cancer diagnosis.

^e^ Includes individuals who are widows.

**Figure.**
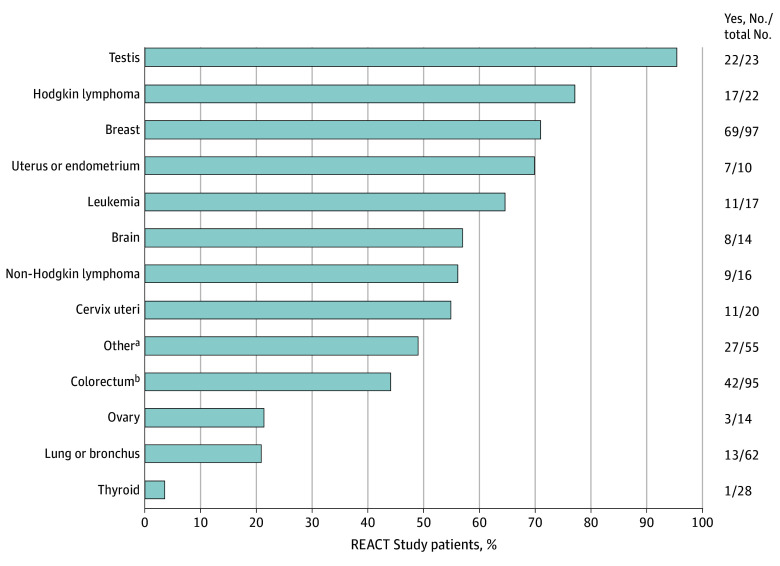
Patterns of Patient-Reported Discussions on Fertility Preservation by Cancer Site (REACT [Reproductive Health After Cancer Diagnosis & Treatment] Study) Proportions of patients across early-onset cancer sites who reported having had a discussion on fertility preservation before treatment initiation. ^a^Cancer sites each with fewer than 10 total cases were included in the other cancers group. These sites include anus, anal canal, and anorectum; appendix; bones and joints; esophagus; kidney and renal pelvis; liver and intrahepatic bile duct; melanoma (skin); myeloma; pancreas; prostate; salivary glands; soft tissue; urinary bladder; vulva; other nervous system; head and neck; stomach; and gastrointestinal stromal tumors. ^b^Colorectum excludes appendiceal cancers.

## Discussion

In this cross-sectional study, we observed that half of the patients did not report having an FP discussion with their health care professional before early-onset cancer treatment. Our study is unique in that it does not focus on what is documented in the electronic medical record (EMR) or on health care professional self-reported practices regarding FP discussions, but, rather, it is patient centered by asking patients what they experienced, heard, and discussed during their clinical encounters with their cancer care team. However, the study of patients’ perceptions or experiences inherently introduces recall bias. It is possible that patients may not have remembered this discussion—which could be attributable to lack of interest in having children, stress from early-onset cancer diagnosis, or inability to pursue FP.^5^ Thus, if FP was discussed by the health care professional but the patient did not understand or recall this information, our findings may underreport the true prevalence of patient-recalled FP discussions in the clinical setting. This also points to the plethora of pressures on an initial oncology visit and other clinical visits after a new early-onset cancer diagnosis and emphasizes the importance of appropriate timing for this conversation.

Because cancer therapies for gonadal function vary by disease site and therapeutic regimen,^1^ our present work adds to the literature^6^ by exploring patterns of patient-reported FP discussions across cancer types for both males and females of reproductive age. Our analyses were conducted using REACT Study data from a large patient population representing 30 early-onset cancer types. We acknowledge that, as a patient-partnered study, our recruitment strategy during COVID-19 contributed to skewed demographics (eg, insured patients or those with higher socioeconomic status) that limit representation of the overall population. The REACT Study also lacks EMR information to assess clinical factors that may affect FP discussions (eg, disease stage, therapeutic regimen that does not affect fertility, or goal of therapy). Although our findings shed light on limited FP discussions as reported by individuals with early-onset cancers, we were unable to evaluate how differences across health care settings or clinical factors may contribute to these patterns. Overall, our study emphasizes the importance of tailoring effective strategies for delivering concordant reproductive health care to the growing population of patients with early-onset cancer.

## Notes

Supplement 1.
eMethods.
**eFigure.** Composition of the Study Population With Exclusion Criteria
eReferences.

Supplement 2.
Data Sharing Statement
