# Supplementary material for: Patient-Reported Discussions on Fertility Preservation Before Early-Onset Cancer Treatment
Source: JAMA Netw Open. 2024 Nov 12;7(11):e2444540. doi: 10.1001/jamanetworkopen.2024.44540 (PMC11558471; doi:10.1001/jamanetworkopen.2024.44540)
Supplement: Supplement 1. — eMethods. eFigure. Composition of the Study Population With Exclusion Criteria eReferences. [file jamanetwopen-e2444540-s001.pdf]

Supplemental Online Content

Keller SR, Rosen A, Lewis MA, et al. Patient-reported discussions on fertility preservation before early-onset cancer treatment. *JAMA Netw Open*. 2024;7(11):e2444540. doi:10.1001/jamanetworkopen.2024.44540

eMethods.

eFigure. Composition of the Study Population With Exclusion Criteria

eReferences.

This supplemental material has been provided by the authors to give readers additional information about their work.

eMethods.

REACT Study

The REACT Study is a patient-partnered, cross-sectional study of adults who were diagnosed with a first primary cancer between ages 18-49 across 32 cancer types recruited over an 8-week period in 2021.[1] To channel the power of social media and patient networks during the COVID-19 pandemic, REACT partnered with 23 community organizations as well as numerous patient advocacy colleagues. Partners featured IRB-approved language in community newsletters, social media posts and/or outreach initiatives—as well as posted information on their webpages. REACT was approved by the Vanderbilt Institutional Review Board (#211691). No informed consent was required to be collected, as the REACT Study met all requirements (having met 45 CFR 46.104 (d) category (2) for Exempt Review) to be classified as exempt by the IRB.

The REACT questionnaire was self-administered, confidential (deidentified) and completed electronically by participants on the study website. This one-time, 30-minute survey included assessment of sociodemographic, cancer and cancer impact/quality of life, detailed reproductive health and fertility, sexual health and energy balance domains. We used both standardized instruments as well as adapted questions from large scale epidemiologic studies, including the National Health and Nutrition Examination Survey (NHANES) and National Health Interview Survey (NHIS),[2,3] and from reproductive endocrinology/infertility clinical intake forms. To reduce the possibility of spam or abuse from automated software programs ('bots') and malicious software that could enter false data into our study, we enabled a Google reCAPTCHA feature.

Study data was collected and managed using REDCap electronic data capture tools.[4,5] Any survey response left blank was categorized as "Not reported".

Study Population

Among REACT participants, we limited our population herein to individuals who responded to a question [source: NIH/NCI AYA Health Outcomes and Patient Experience (HOPE) Study, Follow-Up Survey][6-8]: *Did a healthcare professional involved in your cancer care talk with you about options to preserve your fertility (e.g., sperm banking or freezing of eggs, embryos, or ovarian tissue) before you started cancer treatment?* Participants who did not provide a response (n=4) or who answered: "*I don't remember*" (n=32) were excluded from further evaluation (*95.0% response rate*; eFigure). Cases diagnosed before 2013 (n=89) and females diagnosed with a first primary cancer at the age of 43+ years (n=125) were also excluded. Our final study population was comprised of 473 individuals with early-onset cancer.

Statistical Analysis

Patient-reported variables of interest included fertility preservation discussion (yes, no), age (years) and year at first primary cancer diagnosis, sex assigned at birth (female, male), gender identity (female, male, non-binary, prefer to self-describe), race and ethnicity (non-Hispanic white, other), marital status (single, married or living with partner, divorced or separated), insurance coverage (yes, no) and education level (less than 4-year degree, college graduate/4-year degree, Master's or doctoral/professional school degree) at the time of cancer diagnosis, country of residence (United States,

other), and first primary cancer site (anus, anal canal and anorectum; appendix; bones and joints; brain; breast; cervix uteri; colorectum; esophagus; Hodgkin lymphoma; kidney and renal pelvis; leukemia; liver and intrahepatic bile duct; lung and bronchus; melanoma (skin); myeloma; non-Hodgkin lymphoma; ovary; pancreas; prostate; salivary glands; soft tissue; testis; thyroid; urinary bladder; uterus/endometrium; vulva; other nervous system; head and neck; stomach; and gastrointestinal stromal tumors (GIST)). This article was presented in accordance with the STROBE guidelines for cross-sectional studies. All data were analyzed using SAS version 9.4 statistical software (SAS Institute, NC). All tests were two-sided (unless otherwise specified), with $P<0.05$ considered to be statistically significant.

eFigure. Composition of the Study Population With Exclusion Criteria. REACT, Reproductive Health After Cancer Diagnosis and Treatment; N, number; ASCO, American Society for Clinical Oncology.

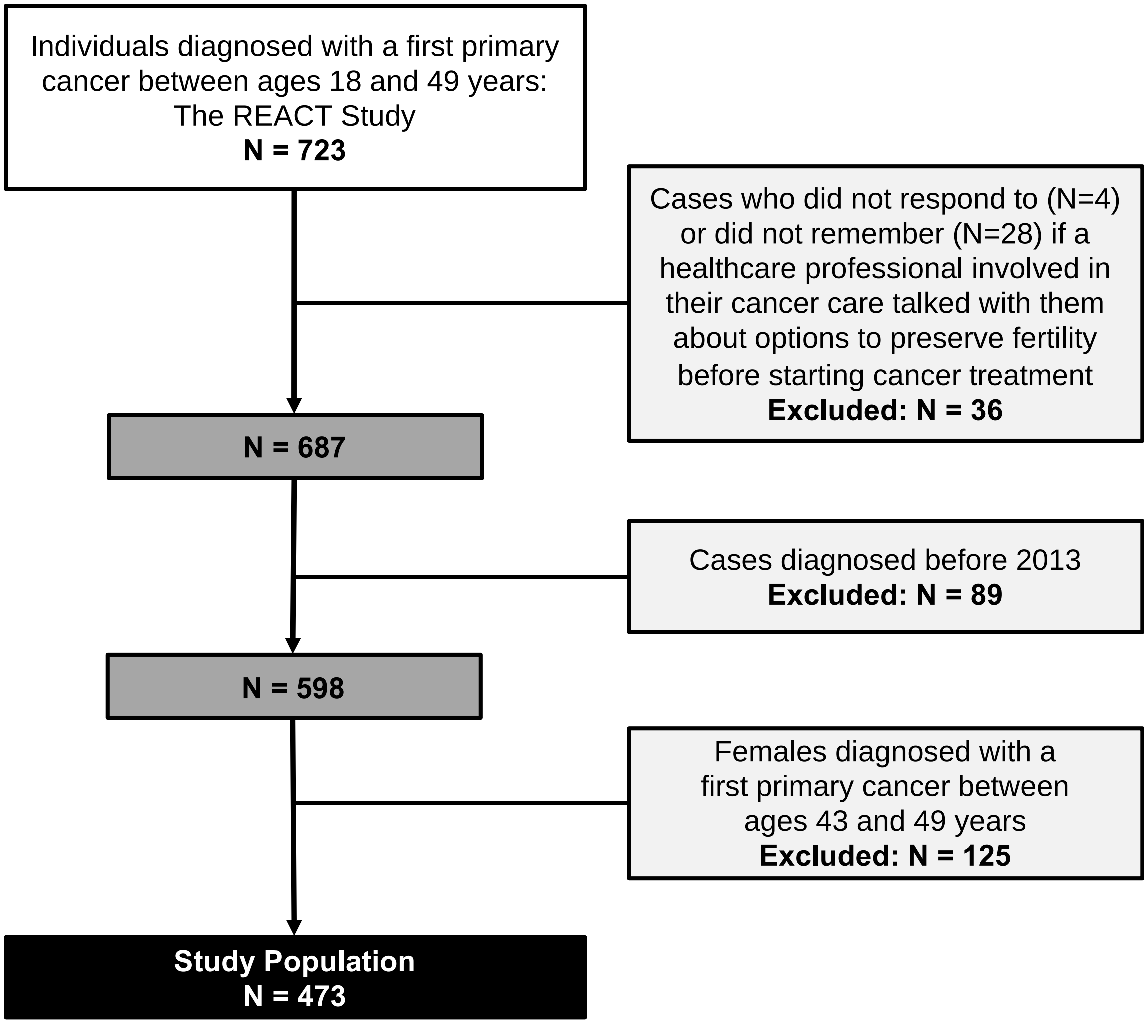

eReferences.

1. Reproductive Health After Cancer Diagnosis and Treatment: The REACT Study. Vanderbilt University Medical Center, Vanderbilt-Ingram Cancer Center. Accessed September 1, 2024, https://www.vumc.org/thereactstudy/home

2. Centers for Disease Control and Prevention (CDC). National Center for Health Statistics (NCHS). National Health and Nutrition Examination Survey Questionnaires. Hyattsville, MD: U.S. Department of Health and Human Services, Centers for Disease Control and Prevention, 2022. https://wwwn.cdc.gov/nchs/nhanes/continuousnhanes/questionnaires.aspx?BeginYear=2019

3. Blumberg SJ, Parker JD, Moyer BC. National Health Interview Survey, COVID-19, and Online Data Collection Platforms: Adaptations, Tradeoffs, and New Directions. *Am J Public Health*. Dec 2021;111(12):2167-2175. doi:10.2105/ajph.2021.306516

4. Harris PA, Taylor R, Thielke R, Payne J, Gonzalez N, Conde JG. Research electronic data capture (REDCap)--a metadata-driven methodology and workflow process for providing translational research informatics support. *Journal of biomedical informatics*. Apr 2009;42(2):377-81. doi:10.1016/j.jbi.2008.08.010

5. Harris PA, Taylor R, Minor BL, et al. The REDCap consortium: Building an international community of software platform partners. *Journal of biomedical informatics*. 2019/07/01/ 2019;95:103208. doi:https://doi.org/10.1016/j.jbi.2019.103208

6. Harlan LC, Lynch CF, Keegan TH, et al. Recruitment and follow-up of adolescent and young adult cancer survivors: the AYA HOPE Study. *Journal of cancer survivorship : research and practice*. Sep 2011;5(3):305-14. doi:10.1007/s11764-011-0173-y

7. Smith AW, Keegan T, Hamilton A, et al. Understanding care and outcomes in adolescents and young adult with Cancer: A review of the AYA HOPE study. *Pediatr Blood Cancer*. Jan 2019;66(1):e27486. doi:10.1002/pbc.27486

8. National Cancer Institute Healthcare Delivery Research Program. Adolescent and Young Adult Health Outcomes and Patient Experience Study (AYA-HOPE) Survey Instruments. National Institutes of Health. Updated April 19, 2024. Accessed September 1, 2024, https://healthcaredelivery.cancer.gov/aya/survey.html
