# Supplementary material for: Patient-Reported Discussions on Fertility Preservation Before Early-Onset Cancer Treatment
Source: JAMA Netw Open. 2024 Nov 12;7(11):e2444540. doi: 10.1001/jamanetworkopen.2024.44540 (PMC11558471; doi:10.1001/jamanetworkopen.2024.44540)
Supplement: Supplement 2. — Data Sharing Statement [file jamanetwopen-e2444540-s002.pdf]

Data Sharing Statement

Keller. Patient-Reported Discussions on Fertility Preservation Before Early-Onset Cancer Treatment. *JAMA Netw Open*. Published November 12, 2024.
doi:10.1001/jamanetworkopen.2024.44540

Data
Data available: Yes
Data types: Data dictionary/codebook, data set
How to access data: REACT Study data are available from the corresponding author and Principal Investigator (A.N.H.) upon reasonable request and as described on the REACT Study website (https://www.vumc.org/thereactstudy/home).
When available: With publication

Supporting Documents
Document types: None

Additional Information
Who can access the data: Researchers whose proposed use of the data has been approved.
Types of analyses: For any purpose.
Mechanisms of data availability: Researchers will be asked to prepare a proposal for scientific feasibility and statistical review. For more information, please reach out to the Principal Investigator of the REACT Study, Dr. Andreana Holowatyj, andreana.holowatyj@vumc.org.
